# Clinical sign and symptom of primary vitreoretinal lymphoma short-time after retinal reattachment surgery: a case report

**DOI:** 10.1186/s12348-022-00283-5

**Published:** 2022-02-05

**Authors:** Marjan Imani Fooladi, Abdulrahim Amini, Hamid Riazi-Esfahani, Nazanin Ebrahimiadib, Mohammadkarim Johari, Fariba Ghassemi

**Affiliations:** 1 Retina service, Farabi eye hospital, Tehran University of medical sciences, Tehran, Iran; 2 Department of Ophthalmology, School of Medicine, Hormozgan University of medical sciences, Bandar Abbas, Iran; 3 Poostchi Ophthalmology Research Center, Department of Ophthalmology, School of Medicine, Shiraz University of Medical Sciences, Shiraz, 7134997446 Iran

**Keywords:** Primary vitreoretinal lymphoma, Retinal detachment, Uveitis

## Abstract

**Purpose:**

To describe a case of primary vitreoretinal lymphoma (PVRL), initially presented after successful repair of rhegmatogenous retinal detachment (RRD).

**Case presentation:**

A 65-year-old man underwent pars plana vitrectomy with silicone oil tamponade for total RRD with grade C proliferative vitreoretinopathy in the right eye. Ten months after silicon oil removal, the patient presented with weakened vision, and multiple small yellow sub-retinal elevations was observed in fundus examination and optical coherence tomography (OCT). A cytopathologic examination of the vitreous showed lymphoid cell infiltration with nuclear atypia, which is strongly indicative of malignant lymphoma. Subretinal lesions continued with no noticeable improvement after 9 sessions of 400 microgram methotrexate therapy.

**Conclusion:**

We identified the presentation of PVRL in a vitrectomized eye and the response to treatment in this article.

## Introduction

Primary CNS lymphoma (PCNSL) is a large B-cell non-Hodgkin’s lymphoma of the brain and it is estimated to be responsible for 4–6% of primary brain tumors. Primary vitreoretinal lymphoma (PVRL) is a subtype of PCNSL, which typically occurs in older population [1]. Most patients with PVRL present with vitreous cells masquerading a posterior uveitis. Another characteristic clinical picture of PVRL is sub-retinalor sub-retinal pigmented epithelium (RPE) infiltrations [1–3].

## Case report

A 65-year-old man was referred to the eye clinic with the chief complaint of decreased vision in his right eye for about one month. He had history of type 2 diabetes mellitus (DM) from 12 years ago, which was controlled with oral hypoglycemic drugs (metformin 1500 mgr /day). Patient had an uncomplicated cataract surgery three years ago in this eye. The best-corrected visual acuity (BCVA) was light perception (LP) and 6/6 in right and left eye, respectively. In fundus examination, a total RRD with supra-temporal retinal tear with grade C proliferative vitreoretinopathy (sub-retinal band) was detected in the right eye, and there was no sign of diabetic retinopathy in either eye. Patient underwent 23-G pars plana vitrectomy with silicone oil tamponade. After 4 weeks, his vision improved to 2/6 with + 6 diopter correction. Four months later, silicone oil was removed and his vision reached 4/6.

**Fig. 1:**
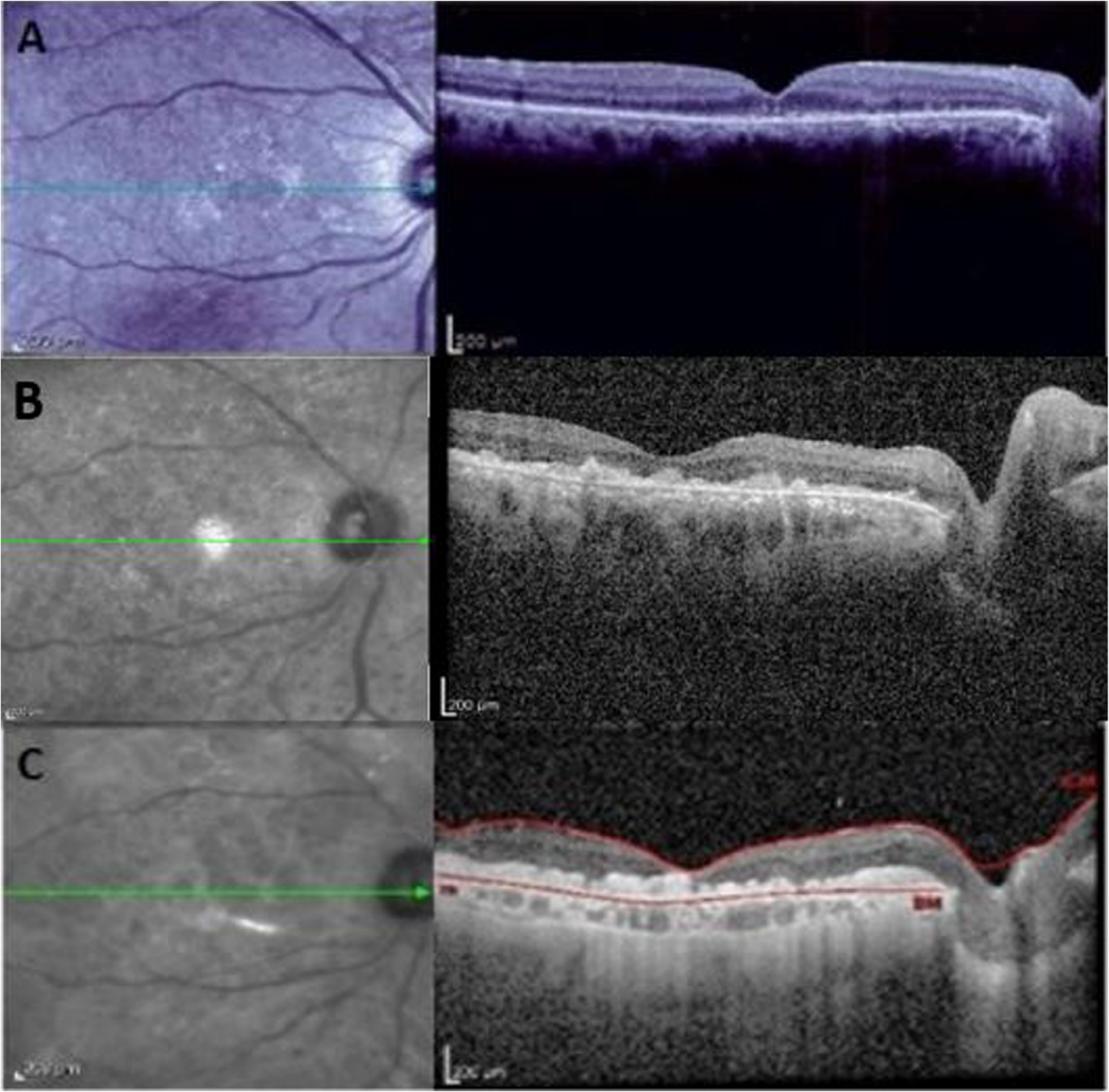
Optical coherence tomography of right eye, (**A**) revealed attached retina with attenuation of the ellipsoid zone (EZ), two vertical hyper reflective lines (Arrow) are seen between the outer retinal layers of the retina and EZ at the foveola. **B** Vitreous opacity obscured retinal details although, irregularity of the ellipsoid zone and outer retinal layers and multiple confluent subretinal and sub-RPE hyper reflective nodular lesions are seen, (**C**) the vitreous infiltration was improved, but the sub retinal and sub-RPE infiltrations were persist

Optical coherence tomography (OCT), in the first week after silicone oil removal, (Fig. 1A) demonstrated an attached retina with attenuation of the ellipsoid zone (EZ) as well as two hype- reflective vertical lines connecting the outer retinal layers to EZ in the fovea region. Some subretinal elevations resembling drusen were also present in nasal macula area. Left eye examination was unremarkable.

**Fig. 2:**
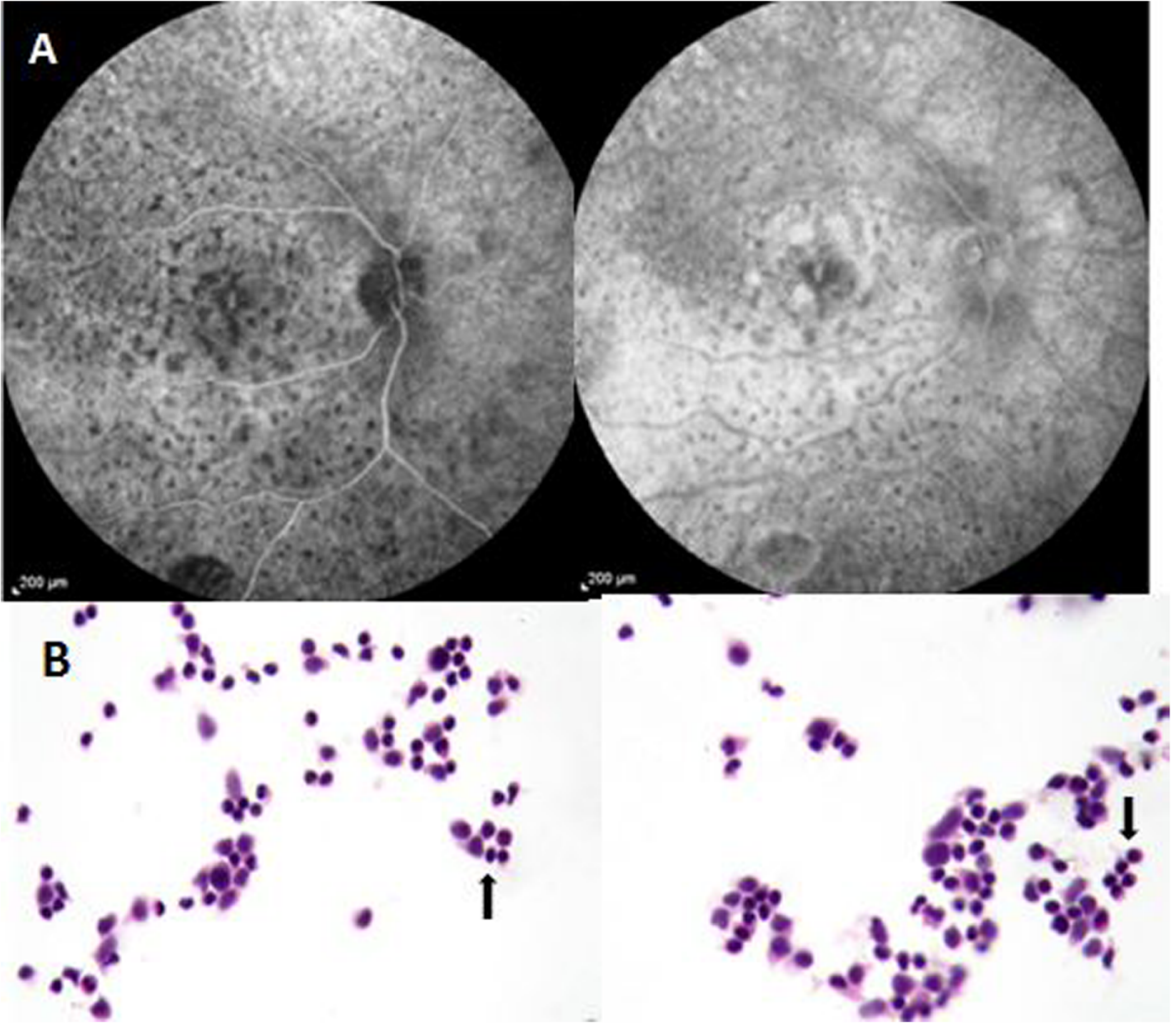
Fluorescein angiography (FA) of right eye early and late phase which shows diffused multiple hypofluorescence dots in the form of a leopard-spot pigmentation within arteriovenous phase. **A** Cytopathology of the vitreous specimen (stained by H&E method) revealed infiltration of lymphoid cell with higher nucleus-cytoplasm ratio and nuclear atypia (Arrows) suggestive of malignant lymphoma. **B**

Ten months later, the patient presented with decreased vision in this eye again. BCVA dropped to hand motion. The intraocular pressure was within normal range in both eyes. Slit lamp examination of the right eye showed diffuse fine corneal keratic precipitates with 1+ cells in anterior chamber. Notably, the anterior vitreous was moderately opaque with + 3 cells infiltrated according to the Standardization of Uveitis Nomenclature (SUN) Working Group grading for intraocular inflammation [4]. In fundus examination, multiple small yellow sub-retinal deposits were visible in the posterior pole, and a mild peripheral serous retinal detachment was observed. OCT revealed multiple confluent sub retinal and sub-RPE hyper reflective nodular lesions with extension to inner retina and complete disappearance of EZ (Fig. 1B). Fluorescein angiography (FA) showed diffused multiple round hypofluorescent lesions in the form of leopard-spot pigmentation during early and later phases of the angiogram. Some deposits in the macular area stained and some pointy leakages were observed in later phases (Fig. 2A). Left-eye examination was unremarkable.

Systemic work-up for infectious and inflammatory etiologies including anti-toxoplasma antibody, tuberculin skin test, Veneral disease research laboratory test (VDRL), FTA-ABS antibody, antineutrophil cytoplasmic antibodies (ANCA), antinuclear antibody (ANA), rheumatoid factor (RF), C-reactive protein (CRP), erythrocyte sedimentation rate (ESR), serum angiotensin enzyme (ACE), and chest x-ray revealed negative results.

Diagnostic vitreous tap was performed. Cytopathology examination of the vitreous specimen revealed infiltration of atypical lymphoid cells with large nuclei to cytoplasm ratio suggestive of malignant lymphoma (Fig. 2B).

Spiral CT-scans of the abdomen, pelvis, neck and thorax showed normal results. Magnetic resonanceimaging (MRI) of the brain noted mild cortical brain atrophy with no sign of PCNSL lymphoma.

The patient was scheduled for intravitreal chemotherapy with methotrexate (MTX) at a dose of 400 μg in 0.1 mL twice per week for 4 weeks, and then eight weekly followed by nine monthly injections. One month after the beginning of the intravitreal injections, no significant change happened in the sub retinal and sub-RPE infiltrations, and the vision was LP and did not improve. After one year, the vision improved to hand motion, vitreous opacity disappeared, and anterior vitreous cells were decreased to trace. However, at the OCT, intraretinal and sub-RPE hyper reflective materials remained (Fig. 1C), so the patient was considered as partially MTX resistance, thereby being scheduled for monthly intravitreal rituximab (1 mg/0.1 ml) injection with periodic brain MRI.

## Discussion

Diagnosis of intraocular lymphoma requires a high degree of clinical suspicion [1]. Due to different treatment approach and effect on survival, differentiating this entity from ocular inflammation is of utmost importance. In our case, ten months prior to the diagnosis of PVRL, there were two OCT signs that, in a retrospective evaluation, were suggestive for the disease when it was still subclinical. The first sign was two hyper-reflective lines with vertical orientation from outer nuclear layer and EZ in the foveola. These vertical lines may be a precursor to sub-RPE infiltrations, and some may be a relation between retinal vessels involvement and sub-RPE deposits, according to some theories [5] Deak et al. hypothesized that these vertical lines may represent early microinfiltrates originating from retinal capillaries, which cannot be recognized in fundus examination. They observed these lines in more than half of their cases. It is worth noting that, sometimes orientation of these lines in relation to OCT B-scans may be oblique, and these lines may appear in inner retina while the rest of B-scans illustrate the line in outer retina. The second OCT sign in our case was drusenoid lesions in macular area, which may be interpreted as aging process or early stages of dry type age-related macular degeneration. Other OCT features have been reported in the literature for PVRL including infiltrations in the inner layers of the retina, discrete nodules of hyperreflective foci in the subretinal space, and confluent bands of hyperreflective foci in the subretinal or sub-RPE space [5, 6]. Based on a case series by Barry et al., each of these OCT features was observed in less than one third of cases [6]. The gold standard test for diagnosis of PVRL is histopathologic investigation of or chorioretinal specimen [7].

The origin of atypical B lymphocytes inside the eye is still questioned in this disease [8]. In addition to typical features of vitreous and subretinal and sub-RPE infiltration, other rare ocular presentations reported for PVRL are vitreous hemorrhages, retinal vasculitis, optic nerve infiltration, and rarely serous retinal detachment [9]. Despite having had RRD surgically repaired, our patient’s PVRL appearance was typical. Therefore, it seems that although previous retinal surgery may not alter the typical signs of PVRL, it may alter the treatment response. Malignant lymphocytes infiltrated the vitreous cavity and anterior segment in our case. It has been postulated that in some patients with empty vitreous, such as vitrectomized eyes, anterior segment manifestations such as corneal edema and KPs may occur more frequently [10].

Treatment is controversial when there is isolated ocular involvement. Local treatment with intravitreal methotrexate (MTX) (400 μg/0.1 cc) is the preferred strategy [1]. It is known that the half-life of the injected drug in the vitreous may be much shorter in a vitrectomized eye. Lee et al. demonstrated the enhanced clearance (more than 10 times) of vascular endothelial growth factors (VEGFs) in vitrectomized eyes [11]. Also, it was stated that aberrant multidrug resistance-related protein (MRP), reduced folate carrier (RFC), and folate binding protein (FBP) expression in the human leukemia cells’ membrane, which might have occurred after multiple intravitreal MTX injections, could disrupt the maintenance of cellular folate homeostasis. They also change the transport of drug across the cells that contribute to resistance following repeated intravitreal injections [12, 13].

As the drug has better access to the vitreous compared to the subretinal space, it could justify the differences therapeutic responses between vitreous malignant cells compared to subretinal infiltrations in our patient. These may explain the reasons why our patient did not respond adequately to the applied treatment.

In these cases, alternative approaches such as ocular irradiation or more frequent injections or higher doses of MTX as well as applying other cytotoxic agent such as rituximab, thiotepa,melphalan or autologous peripheral blood stem cell transplantation may be helpful [14–17]. To validate these approaches, further studies are needed.

This is the first article to explain how PVRL manifests itself shortly after retinal detachment surgery and how it responds to treatment. OCT has an indispensable role in the diagnosis and treatment response which was proved by pathology.

## Conclusions

PVRL is a rare ocular malignancy of older age that commonly masquerades posterior uveitis. Our case suggested that although retinal surgery may not alter the typical sign of PVRL, it may alter the response to treatment with intravitreal chemotherapy. Of note, OCT features such as hyper-reflective vertical lines and subretinal or sub RPE deposits are helpful for a prompt diagnosis.

## Data Availability

Data is available as needed.
